# Hepatitis Delta Virus (HDV) and Delta-Like Agents: Insights Into Their Origin

**DOI:** 10.3389/fmicb.2021.652962

**Published:** 2021-06-21

**Authors:** Hans J. Netter, Marilou H. Barrios, Margaret Littlejohn, Lilly K. W. Yuen

**Affiliations:** ^1^Victorian Infectious Diseases Reference Laboratory (VIDRL), Melbourne Health, The Peter Doherty Institute, Melbourne, VIC, Australia; ^2^School of Science, Royal Melbourne Institute of Technology (RMIT) University, Melbourne, VIC, Australia; ^3^The Peter Doherty Institute, University of Melbourne, Melbourne, VIC, Australia

**Keywords:** hepatitis delta virus, delta-like agent, satellite virus, hepatitis B virus, helper virus, subviral agent

## Abstract

Hepatitis delta virus (HDV) is a human pathogen, and the only known species in the genus *Deltavirus*. HDV is a satellite virus and depends on the hepatitis B virus (HBV) for packaging, release, and transmission. Extracellular HDV virions contain the genomic HDV RNA, a single-stranded negative-sense and covalently closed circular RNA molecule, which is associated with the HDV-encoded delta antigen forming a ribonucleoprotein complex, and enveloped by the HBV surface antigens. Replication occurs in the nucleus and is mediated by host enzymes and assisted by *cis*-acting ribozymes allowing the formation of monomer length molecules which are ligated by host ligases to form unbranched rod-like circles. Recently, meta-transcriptomic studies investigating various vertebrate and invertebrate samples identified RNA species with similarities to HDV RNA. The delta-like agents may be representatives of novel subviral agents or satellite viruses which share with HDV, the self-complementarity of the circular RNA genome, the ability to encode a protein, and the presence of ribozyme sequences. The widespread distribution of delta-like agents across different taxa with considerable phylogenetic distances may be instrumental in comprehending their evolutionary history by elucidating the transition from transcriptome to cellular circular RNAs to infectious subviral agents.

## Life Cycle of HDV and Its Dependence on HBV as Helper Virus

Hepatitis delta virus (HDV) is a unique human pathogen, and has been the only known species in the genus *Deltavirus* (Magnius et al., 2018), but was reclassified in a new family *Kolmioviridae*, genus *Deltavirus* within one new realm *Ribozyviria* [International Committee on Taxonomy of Viruses (ICTV), 2020]. Due to the possession of a circular RNA genome and its mechanism of replication, similarities exist with viroids, which represent a large family of subviral plant pathogens (Flores et al., 2016; Adkar-Purushothama and Perreault, 2019). But HDV is clearly distinguished from the viroids by its larger genome size and the ability to encode a protein. The recent discovery of delta-like agents in various animal species has broadened the views on the evolutionary history of HDV (Wille et al., 2018; Chang et al., 2019; Hetzel et al., 2019; Paraskevopoulou et al., 2020; Bergner et al., 2021; Iwamoto et al., 2021).

The existence of HDV was discovered in 1977 by the identification of a new antigen, the delta antigen (HDAg), in liver biopsies and sera from patients with a severe form of hepatitis B (Rizzetto et al., 1977). Experimental transmission studies (Rizzetto et al., 1980; Ponzetto et al., 1984), then the cloning of the HDV genome (Denniston et al., 1986; Wang et al., 1986; Makino et al., 1987) demonstrated that the HDAg is associated with a separate transmissible agent. HDV is a satellite virus and depends on the human hepatitis B virus (HBV) surface proteins (HBsAg) for packaging, release, and transmission.

**FIGURE 1:**
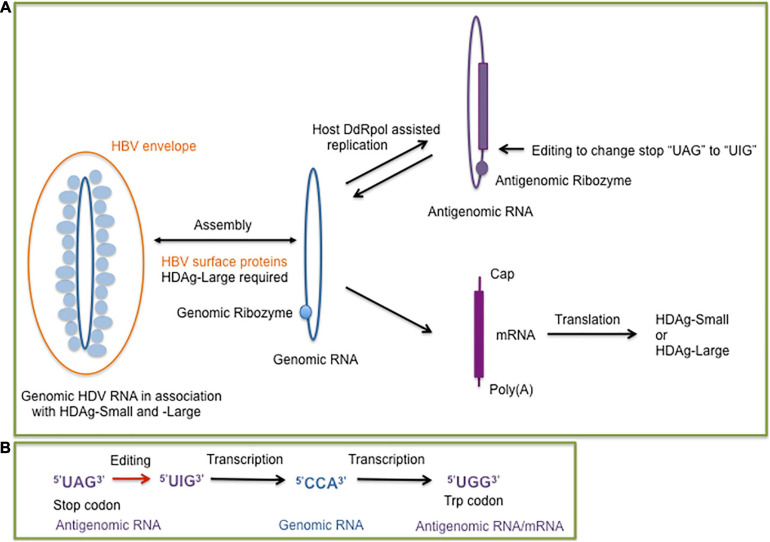
**(A)** Overview of the hepatitis delta virus (HDV) replication cycle supported by the host DNA dependent RNA polymerase (DdRp) to replicate the genomic (blue) and antigenomic (purple) circular single-stranded RNA molecules. The presence of the genomic and antigenomic ribozyme is indicated by filled circles. The genomic RNA serves as a template for the synthesis of the mRNA which encodes the small or large delta antigen (HDAg-Small, HDAg-Large). An editing event at the antigenomic RNA results in the formation of genomic RNAs for the synthesis of mRNA molecules encoding HDAg-Small or HDAg-Large. The dependence of HDV packaging on the hepatitis B virus (HBV) envelope (surface) proteins is indicated. **(B)** Steps of the editing outcome which converts the stop codon of the HDAg-Small open reading frame to a tryptophan codon. The editing of the antigenomic RNA converts the adenosine of the “UAG” stop codon to an inosine, which prefers to pair with cytosine during replication. The edited genomic RNA transcribes into a mRNA with the “UGG” tryptophan codon, which allows a 19 amino acid extension of the open reading frame for HDAg-Large synthesis (Polson et al., 1996).

Extracellular HDV virions exclusively contain genomic HDV RNA, a single-stranded negative-sense and covalently closed circular RNA molecule with a size of 1,668–1,697 nucleotides, depending on the genotype (Le Gal et al., 2017) in contrast to viroids with a size range between approximately 250–400 nucleotides (Flores et al., 2016). HDV does not encode for an RNA-dependent-RNA polymerase (RdRp), but depends on host DNA-dependent RNA polymerases (DdRp) to facilitate RNA-directed RNA synthesis for transcribing and replicating the genome in the cell nucleus (Modahl et al., 2000; Chang et al., 2008; Sureau and Negro, 2016). The negative-sense, genomic RNA is replicated by a rolling circle mechanism to generate its complement, the positive-sense, antigenomic RNA, which serves as a replication intermediate for the synthesis of the genomic RNA (Figure 1A). Both *de novo* synthesized genomic and antigenomic RNA molecules are processed into monomers by intrinsic ribozymes, which are formed by nested double pseudoknot structures (Kuo et al., 1988; Perrotta and Been, 1991; Ferré-D’Amaré et al., 1998; Sureau and Negro, 2016). Monomer synthesis is followed by the formation of circular RNA molecules by intramolecular ligation (Sharmeen et al., 1989; Reid and Lazinski, 2000).

The HDV genomic RNA and its antigenomic complement are highly self-complementary and form unbranched rod-like structures in native conditions with approximately 74% intramolecular base pairing (Kos et al., 1986; Wang et al., 1986). HDV shares the features of a circular self-complementary RNA genome with viroids (Flores et al., 2016; Adkar-Purushothama and Perreault, 2019). Contrary to viroids, HDV generates a mRNA transcript which encodes the HDAg (Hsieh et al., 1990). HDV expresses two HDAg forms, which interact with HDV RNA to form ribonucleoprotein (RNP) complexes (Ryu et al., 1993) but they play different roles during the HDV life cycle. The small form of the HDAg (HDAg-S) is expressed throughout the HDV infection, it is essential for HDV replication and HDV RNA accumulation but does not exhibit a RdRp activity and stimulates DdRp II elongation (Kuo et al., 1989; Hsieh et al., 1990; Yamaguchi et al., 2001). During infection and HDV replication, a site-specific editing event occurs at some antigenomic RNAs, which allows the generation of mRNAs with the UAG stop codon replaced with the tryptophan UGG codon (Figure 1B). The extended HDAg open reading frame by 19 amino acids encodes the large form of HDAg (HDAg-L) (Luo et al., 1990; Polson et al., 1996; Wong and Lazinski, 2002). Following the editing event, HDAg-L accumulates during HDV replication and facilitates HDV assembly in the presence of the HBV HBsAg proteins (Chang et al., 1991). The additional 19 amino acids contain the carboxyterminal “CXXX” sequence motif allowing HDAg-L farnesylation, which is essential for the assembly process (Glenn et al., 1992; Hwang and Lai, 1993; O’Malley and Lazinski, 2005). The farnesyl group facilitates RNP attachment to the membrane of the endoplasmic reticulum (ER), the site of HBsAg synthesis. The proximity of the hepatitis delta RNPs and HBV surface proteins allows the envelopment of the hepatitis delta RNPs at the ER membrane to generate hepatitis delta virions. HDAg-L in the absence of HDV RNA and HDAg-S can be packaged by HBsAg proteins indicating that HDAg-L is the driver of HDV assembly and release (Chang et al., 1991; Chen et al., 1992; Ryu et al., 1992).

HDV depends on the HBV envelope proteins for its life cycle completion and the production of infectious HDV particles. HBV encodes three related surface proteins, the shortest surface protein HBsAg-S is composed only of the S-domain, the middle and large HBsAg proteins (HBsAg-M and HBsAg-L) contain additional N-terminal extensions of the S-domain. In particular, the dual topology of the preS1 domain of HBsAg-L is essential for hepatitis B virion assembly and attachment to the host cell. The preS1 domain of newly synthesized HBsAg-L proteins are on the cytosolic side and interact with the hepatitis B nucleocapsid allowing virion assembly. During the HBV budding process, the HBsAg-L preS1 domain translocates across the viral lipid layer to be surface exposed (Bruss et al., 1994; Ostapchuk et al., 1994), which is essential for the preS1 binding site to be able to engage the viral receptor, “sodium taurocholate cotransporting polypeptide” (NTCP) (Yan et al., 2012). As for HBV, HDV requires the preS1 domain of HBsAg-L for binding to the NTCP receptor (Yan et al., 2012). But in contrast to the HBV morphogenesis, the preS1 domain is not required for the HDV assembly and budding process. The presence of only HBsAg-S proteins is sufficient for HDV assembly and release of HDV particles, but in the absence of the HBsAg-L preS1 domain, they are non-infectious (Sureau et al., 1993). Several HBV envelope S-domains are involved in HDV assembly and secretion including the internal, cytosolic loop and a tryptophan-rich C-terminal sequence (Jenna and Sureau, 1999; Komla-Soukha and Sureau, 2006). Compared to HBV with a diameter of approximately 42 nm, HDV is less dense and slightly smaller in size, approximately 39 nm in diameter (He et al., 1989; Hu and Liu, 2017).

The dynamics of HDV infections following orthotopic liver transplantations provided evidence that HDV can cause subclinical helper-independent or mono-infections (Ottobrelli et al., 1991; Samuel et al., 1995) but HDV viremia requires the HBV helper function (Smedile et al., 1998). The detection of HDAg in the liver in the absence of HBV markers is possibly an indicator of HDV latency in the liver (Mederacke et al., 2012). Consistently, animal models demonstrated that HDV replicates and also persists in helper independent- or mono-infection contexts. The absence of HBV HBsAg or envelope proteins from a closely related mammalian hepatitis B virus, woodchuck hepatitis B virus (WHV), allowed HDV replication but no progression to viral assembly and release (Netter et al., 1993; 1994; Giersch et al., 2014). Studies with the woodchuck animal model and humanized mice showed that HDV during a mono-infection phase could be rescued by inoculating the corresponding WHV or HBV helper virus resulting in HDV viremia (Netter et al., 1994; Giersch et al., 2014). Alternatively, integrated HBV DNA can provide functional HBsAg-L and HBsAg-S transcripts and proteins to facilitate formation of infectious HDV in the absence of HBV replication (Freitas et al., 2014).

A cell culture based study showed that HDV can be pseudotyped with envelope proteins derived from various viruses, including vesicular stomatitis virus (VSV), hepatitis C virus (HCV), and Dengue virus. The VSV and HCV envelope proteins supported the release and assembly of genomic HDV RNA, also depending on HDAg-L farnesyl-mediated targeting of cell membranes similar to the assembly process supported by HBV envelope proteins (Perez-Vargas et al., 2019). The HDV RNPs pseudotyped with HCV envelope proteins and Dengue glycoproteins generated infectious HDV particles supporting entry and replication in human hepatoma cells HuH7.5 and insect C6/36 cell lines, respectively. However, the clinical relevance of these findings remain uncertain. A recent study that involved 323 HCV RNA positive and HBsAg-negative patients could only detect HDV markers in eight HBV core antibody (anti-core) positive patients (evidence of past acute HBV infections) and not among the remaining HBV core antibody negative patients suggesting the occurrence of replicative HDV infections in HCV mono-infected patients is low (Pflüger et al., 2020). A similar study investigating a cohort of 160 Venezuelan patients infected with HCV in the absence of molecular markers for HBV detected two patients with anti-HDAg antibodies, and for one patient low-level circulating HDV RNA (Chemin et al., 2020), also indicating that if HCV provides helper functions, it does not seem to be an effective or potent helper virus. Furthermore, HDV RNA and HDAg have been detected in the salivary glands of patients with a primary Sjögren’s syndrome in the absence of a past or current HBV infection, which leaves the unanswered question of how HDV established an infection in these patients (Weller et al., 2016). Interestingly, Perez-Vargas et al. (2019) confirmed earlier studies that HDV replication is not restricted to human liver cells. HDV replication in the absence of HBV helper function for assembly and release has been reported for human embryonic kidney cells, mouse skeletal muscle cells, and hamster kidney cells (Bichko et al., 1994; Polo et al., 1995; Chang et al., 2005) but HDV replication is restricted in avian cells due to the cytotoxicity of the delta antigen (Chang et al., 2000). The ability of HDV to replicate in different cell types, the recent identificaton of HDV-like agents in diverse vertebrate and invertebrate species (Chang et al., 2019) and the presence of cellular ribozymes with structural similarities to the HDV-ribozyme (Riccitelli and Lupták, 2013) possibly suggests that HDV originates from the cell transcriptome, due to a process related to the biogenesis of cellular circular RNAs found in eukaryotes (Kristensen et al., 2019).

### HDV Genotypes and Delta-Like Agents

Natural HDV infections have only been described in humans, and hence HDV most likely co-evolved with the helper HBV in the human lineage. Experimental transmission of HDV and HBV to chimpanzees (Rizzetto et al., 1980), and the acceptance of mammalian hepatitis B viruses (genus *Orthohepadnavirus*), such as the woodchuck hepatitis B virus (WHV) as alternative helper viruses for HDV assembly and transmission allowed the establishment of animal models to study HDV replication and pathogenesis (Ponzetto et al., 1984; Netter et al., 1994; Gerin, 2001; Aldabe et al., 2015).

**FIGURE 2:**
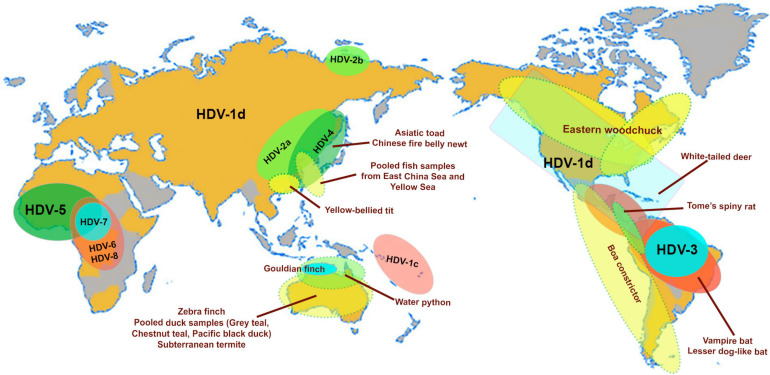
Map highlights the geographical distribution of HDV genotypes (HDV1-8) and subgenotypes (HDV1c, 1d; HDV-2a, b) and the worldwide distribution of animal hosts of identified delta-like agents (Asiatic toad–*Bufo gargarizans*; Newt–*Cynops orientalis*; Yellow-bellied tit–*Pardaliparus venustulus*; Gouldian finch–*Erythrura gouldiae*; water pyton–*Liasis mackloti*; Zebra finch–*Taeniopygia guttata*; subterranean termite–*Schedorhinotermes intermedius*, lesser dog-like bat–*Peropteryx macrotis*; vampire bat–*Desmodus rotundus*; Tome’s spiny rat–*Proechimys semispinosus*; white-tailed deer–*Odocoileus virginianus*; Eastern woodchuck–*Marmota monax* (Paraskevopoulou et al., 2020; Bergner et al., 2021; Iwamoto et al., 2021). The pooled fish samples contain species from the classes Actinopterygii, Chondrichthyes and Agnatha (Chang et al., 2019), and the waterfowl samples were collected from gray teal (*Anas gracilis*), chestnut teal (*Anas castanea*), and pacific black duck (*Anas superciliosa*) (Wille et al., 2018). The map is adapted from Le Gal et al. (2017).

Eight distinct HDV genotypes have been documented in human populations. The HDV genotypes differ in their genomic sequence by 19–40% (Le Gal et al., 2017), and can be further sub-categorized into two to four subgenotypes with the exception of HDV genotype 3 (HDV-3). Their global distribution is geographically distinct except for HDV-1d (Figure 2; Le Gal et al., 2017). The subgenotype HDV-1d is prevalent worldwide, and represents the dominant HDV strain in Europe and North America. In contrast, HDV-1a and -1b are predominantely found in Africa and the Middle East, while HDV-1c is the dominant strain in the Western Pacific region (Han et al., 2014). The subgenotypes HDV-2a, HDV-4a, and HDV-4b are mainly distributed in Southeast Asia, China, Japan, and Taiwan; HDV-2b in Russia (Siberia). HDV-3 is mainly located in the Northern part of South America, and HDV-5 to HDV-8 are found in Africa (Le Gal et al., 2017).

Based on full-length genome and deduced HDAg amino acid sequences, HDV-3 seems to be the most distantly related human HDV genotype and has the lowest similarity score when compared to the other HDV genotyes (Casey et al., 1993). The distant genetic relationship of HDV-3 to the other genotypes is further indicated by the inability of the HDAg encoded by HDV-3 to support replication of HDV-1, and vice versa (Casey and Gerin, 1998). Depending on the genotype, HDV can cause disease manifestations of different severity, HDV-2 is normally associated with a milder disease progession than HDV-1. Infections with HDV-3 in combination with HBV genotype F, which is the predominant HBV in the northern part of South America are associated with an enhanced risk of fulminant hepatitis (Casey et al., 1996). With the presence of HDV genotypes 5–8, and sub-genotypes 1a and 1b, central Africa around Cameroon is possibly the main site of HDV diversication resulting in an ancient radiation of the African lineages (Radjef et al., 2004; Le Gal et al., 2017).

Advances in metagenomics have lead to the discovery of delta-like agents from the transcriptome libraries generated for a number of non-human vertebrates and invertebrates in recent years (Wille et al., 2018; Chang et al., 2019; Hetzel et al., 2019; Paraskevopoulou et al., 2020; Bergner et al., 2021; Iwamoto et al., 2021). Of the large number of libraries screened in these studies to date, delta-like agents have been identified in at least nine taxa including birds (gray teal, chestnut teal, Pacific black ducks, zebra finch, Gouldian finch, canary, yellow-bellied tit), termite (subterranean termite), fish (pooled sample from multiple species), toad (Asiatic toad), newt (Chinese fire belly newt), snake (boa constrictor, water python), rat (Tome’s spiny rat), woodchuck (Eastern woodchuck), bat (common vampire bats, lesser dog-like bat), and deer (white-tailed deer). Surprisingly, delta-like agents were not detected in non-human primates indicating that HDV is the only known representative infecting the order Primates (Bergner et al., 2021). The natural habitats of the taxa mentioned above in relation to the geographical location of the human HDV genotype and subgenotypes are shown in Figure 2. The ability for these non-human delta-like agents to transmit and replicate in nature remain unclear, but it has been shown that the rodent delta-like agent is able to replicate *in vitro* (Paraskevopoulou et al., 2020) and that the bat delta-like agent can transmit to other members of the same colony based on prevalence studies of bat colonies (Bergner et al., 2021). As analyzed for delta-like agents identified in the Eastern woodchuck (*Marmota monax*), canary (*Serinus canaria*), Zebra finch (*Taeniopygia guttata*) and white-tailed deer (*Odocoileus virginianus*), the read depths of predicted transcribed regions (the coding region for the delta-like antigen) were greater than those of other genomic regions indicating that most delta reads were derived from delta mRNAs suggesting that the novel delta-like agents replicate in their hosts (Iwamoto et al., 2021). Nonetheless, no definitive helper virus for assembly and transmission of these delta-like agents within their hosts have been identified to date. It has been suggested that an alternative supply of envelope proteins for the non-human delta-like agents may be provided by endogenous viral elements (EVEs), which are encoded within the host genome, or they may utilize strategies distinct from those employed by HDV (Iwamoto et al., 2021). HBV EVEs have been identitifed in the genome of birds of the order *Passeriforme* including zebra finch (Gilbert and Feschotte, 2010) and budgerigars of the order *Psittaciformes* (Cui and Holmes, 2012), however *in vitro* studies confirmed that the zebra finch delta-like agent, also the woodchuck delta-like agent, did not use the small HBsAg supplied *in trans* to generate infectious virions (Iwamoto et al., 2021). Interestingly, the Eastern woodchuck (*Marmota monax*) is the host for the woodchuck hepatitis B virus (WHV) which is an efficient helper virus providing the envelope proteins for the assembly of infectious HDV in the woodchuck animal model (Netter et al., 1994; Gerin, 2001). No natural HDV infection has been reported in this animal population, and there is no evidence that HBV supports packaging and release of infectious woodchuck-derived delta-like agents (Iwamoto et al., 2021). This strongly indicates that the presence of a potential helper virus and a delta-like agent does not necessarily drive the emergence of a satellite RNA virus. This finding supports the proposition that non-human delta-like agents may use a novel mechanism for assembly and transmission.

The phylogenetic relationships between HDV and the delta-like agents have been consistent between studies (Paraskevopoulou et al., 2020; Bergner et al., 2021; Iwamoto et al., 2021), though minor clustering variations do occur between the full-genome and HDAg phylogenetic trees. Based on full-genome phylogenetic trees, HDV and the non-human delta-like agents formed two distinct clusters (Paraskevopoulou et al., 2020). In this study, the delta-like agent genomes further clustered into two distinct groups (the snake and rodent delta-like agents vs. the newt, toad, fish, termite, and duck delta-like agents). Interestingly, the HDAg amino acid sequences of the newly discovered deer (*Odocoileus virginianus*), woodchuck (*Marmota monax*), and one of the bat (*Desmodus rotundus*) genotypes of delta-like agents were found to form a sister clade with the human HDV (Bergner et al., 2021). The HDAg of these delta-like agents shared a common ancestor with the HDAg of a second bat (*Desmodus rotundus*) genotype, rodent (*Proechimys semispinosus*), snake (*Boa constrictor*) and duck delta-like agents, while the remaining delta-like agents formed a more distal clade (derived from fish, newt and toad). However, despite the low level of identity between the HDAg protein sequences of delta-like agents from different taxa (14–67%) (Iwamoto et al., 2021), no clear distinction between the vertebrate and invertebrate delta-like agents could be detected by phylogenetic analyses. In addition to the finding that the bat and rodent delta-like agents were paraphyletic, cophylogenetic analyses between delta-like agent and host trees did not support the theory that delta-like agents had co-speciated with their hosts but may have evolved by host shifting (Bergner et al., 2021; Iwamoto et al., 2021).

### Hypotheses of Origin

(i) Delta-Like Agents and Cellular Circular RNAs as Potential Precurors of Subviral Agents.

**FIGURE 3:**
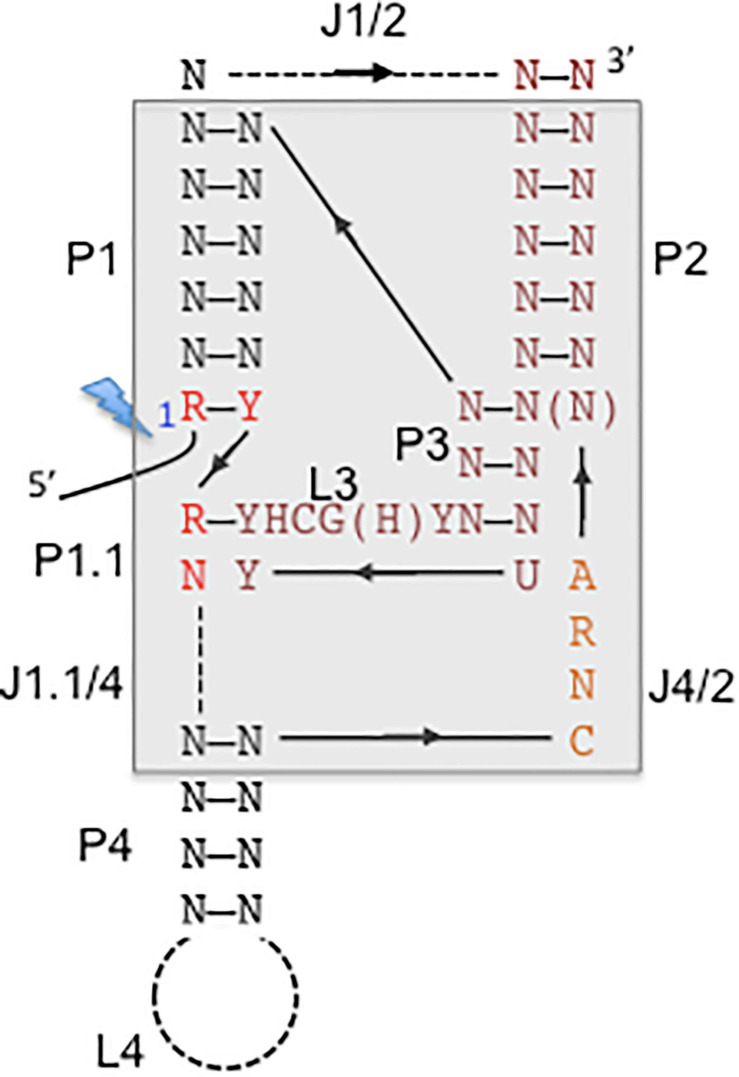
Consensus secondary structure of hepatitis delta virus (HDV)-like ribozymes. The catalytic core is boxed with gray background. The base-paired regions are numbered with P1–P4, which are linked by single-stranded regions, J1/2, J4/2, and J1.1/4. L stands for single-stranded loop regions. Depending on the ribozyme, J1/2 region and P4 helix can be expanded indicated by the dashed lines allowing sequence length variations. The solid line represents direct connections without nucleotide insertions. The arrows indicate the direction of the strands from 5′ to 3′. The 5′ end highlighted by a lightning bolt marks the 5′-OH end after self-cleavage. N stands for any nucleotide, R for purine, Y for pyrimidine, H for adenine, cytidine or uracyl. The figure is adapted from Ferré-D’Amaré et al. (1998) and Webb and Lupták (2011).

Viroids were the first circular RNA discovered (Sänger et al., 1976), then followed by the discovery of cellular circular RNA located in the cytoplasm of eukaryotic cells (Hsu and Coca-Prados, 1979). Circular RNAs (circRNAs) can be generated by alternative splicing or backsplicing and results in the formation of different circRNA species composed of only exon sequences (exonic circRNAs), circRNAs with both intron and exon sequences (exon-intron circRNAs), and circRNAs with only intronic sequences (ciRNAs). Alternatively, the formation of circRNA can be assisted by ribozymes, such as the HDV-related ribozymes, which have a widespread occurence in eukaryotic genomes (Figure 3). CircRNAs with hammerhead ribozymes have been identified in eukaryotic genomes, and it has been hypothesized that they have given origin to infectious circRNAs (De la Pena and Cervera, 2017). The covalently closed ring structure confers stability avoiding exonuclease-mediated degradation (Kristensen et al., 2019; Lasda and Parker, 2014). An abundance of circRNAs have been identified in divergent animal and plant species. For human fibroblast cells it was reported that circRNA molecules originate from more than 14% of transcribed genes (Jeck et al., 2013). The biogenesis of circular cellular RNAs is antagonized by the RNA adenosine deaminase (ADAR) by editing endogenous double-stranded (ds) RNA sequences. ADAR faciliates in combination with an ATP-dependent RNA helicase A (DHX9) the melting of dsRNA stems and hence prevents the looping of intron sequences and the formation of circRNA (Ivanov et al., 2015). Interestingly, the circular HDV RNA depends on ADAR editing for the completion of the viral reproductive cycle. HDV antigenomic RNA requires editing to generate mRNAs with an extended open reading for the synthesis of HDAg-L, which is essential for the HDV envelopment by the HBV surface proteins. ADAR targets the partially double-stranded editing site of the circular HDV antigenomic RNA (Polson et al., 1996) to ultimately convert the amber stop codon to a tryptophan codon (Figure 1B). HDV editing by ADAR which targets partially double-stranded RNA molecules and RNA loop structures points together with the widespread distribution of the HDV-like ribozyme and the prevalence of circRNA molecules in eukaryotes toward a long evolutionary history of HDV.

The recent discovery of delta-like agents in metagenomic samples from birds and snake, then in fish, amphibians and invertebrates demonstrates that the RNA genomes are highly divergent (Wille et al., 2018; Chang et al., 2019; Hetzel et al., 2019; Bergner et al., 2021; Iwamoto et al., 2021). The delta-like agents share common features with HDV, the circular genome with a size of approximately 1.7 kb (between 1,547 and 1,735 nucleotides), self-complementarity to fold into rod-like structures, and the presence of an open reading frame (ORF) (Chang et al., 2019). Delta-like agents identified in tissues from snakes (Hetzel et al., 2019), a rodent species (*Proechimys semispinosus*) (Paraskevopoulou et al., 2020) and in combined oropharangeal and cloacal samples from teals and ducks (Wille et al., 2018) contain HDV-like ribozymes (Table 1). Importantly, the presence of delta-like agents does not seem to be associated with members of the hepatitis B virus family indicating that HDV as a delta-agent possibly co-evolved with HBV in humans and optimized the assembly efficacy and release. The HBV envelope proteins provide hepatocyte-specific tropism (Bonino et al., 1986; Yan et al., 2012) but the HDV genome retained the ability to replicate in non-liver cells (Bichko et al., 1994; Polo et al., 1995; Chang et al., 2005). In contrast, the snake delta-like agent (sDLA) does not exhibit strict assembly requirements as clearly demonstrated for HDV and its dependence on the HBV envelope proteins. The inoculation of boa kidney cells with sDLAs derived from an infected brain homogenate passaged sDLAs in the presence of coinfecting Arenaviruses (Hartmanivirus, Reptarenavirus). Transfection experiments with glycoproteins from Arenaviruses and Orthohantavirus allowed the formation of infectious sDLAs (Szirovicza et al., 2020). The sDLA antigen (sDL-Ag) was identified in different tissues of infected animals, and consistently, sDLA replication similar to HDV replication, is supported by different cell types (Bichko et al., 1994; Polo et al., 1995; Chang et al., 2005; Szirovicza et al., 2020). Rodent DLA (rDLA) was identified in blood samples, not linked to liver tropism, and interestingly, predominantly detected in reproductively active males living in continuous forest sites, suggesting horizontal transmission linked to competitive behavior. The study did not identify a helper virus and transmission is possibly assisted by envelope proteins provided by an unkown agent, or by extracellular vesicles, which include microvesicles and exosomes (Kim et al., 2017; Paraskevopoulou et al., 2020). It was proposed for retroviruses to exploit the exosome exchange for a low efficiency mode of infection (Gould et al., 2003). Hepatitis C virus (HCV)-RNA containing exosomes have been identified for the export of viral RNA to plasmacytoid denritic cells (Dreux et al., 2012), and importantly mediate transmission of HCV between hepatocytes (Ramakrishnaiah et al., 2013). Similarly, HBV virions were detected in extracellular vesicles collected from infected patients (Kakizaki et al., 2018), and HBV RNA in extracellular vesicles from HBV transfected hepatocytes (Kouwaki et al., 2016). CircRNAs are present in exosomes (Kim et al., 2017; Fanale et al., 2018; Veziroglu and Mias, 2020), and hence delta-like agents could be transmitted with low efficiency as proposed for retroviruses according to the Trojan exosome hypothesis (Gould et al., 2003). The exact mechanisms by which RNAs are loaded into exosomes remain unclear but certain motifs and double-stranded stem-loop secondary structure were proposed to be important for packaging (Villarroya-Beltri et al., 2013; Kossinova et al., 2017).

**TABLE 1:** Characteristics of hepatitis delta virus (HDV), delta-like agents, cell-encoded circular RNAs, and plant viroids.

	**HDV**	**Delta-like agents**	**Cell-encoded circular RNA**	**Viroids**	
				**Pospiviroidae**	**Avsunviroidae**
Single-stranded circular RNA	✓	✓	✓	✓	✓
Self-complementarity	✓	✓	Formation of stem/loop structures possible	✓	✓
Unbranched rodlike structure	✓	✓		✓	×
Host DdRpol assisted replication	✓	✓^‡^	Derivatives from splicing events	✓	✓
Ribozyme	✓	✓	With or without ribozyme (hammerhead)	×	✓
HDV ribozyme/HDV-like ribozyme	✓	✓*			×
ORF, encoding of protein	✓	✓	With or without ORF	×	×

DdRpol, DNA-dependent RNA polymerase; ORF, open reading frame; ^‡^delta-like agents do not encode a polymerase, and most likely replication assisted by DdRpol. *Delta-like ribozymes were not identified for delta-like agents derived from the toad (Bufo gargarizans), termite (Schedorhinotermes intermedius), newt (Cynops orientalis) and fish (derived from a pool of fish species, Chang et al., 2019) hosts.

Both the sDLAs and rDLAs studies demonstrated the expression of the corresponding DL-Ags confirming that the ORF is translated (Paraskevopoulou et al., 2020; Szirovicza et al., 2020). Transfection of expression vectors with dimer genomes for delta-like agents derived from woodchuck and Zebra finch resulted in the expression of the corresponding delta-like antigens (Iwamoto et al., 2021). The identified delta-like agents from vertebrates and invertebrates encode DL-Ags including additional in-frame reading frames downstream of the stop codon or in alternative reading frames demonstrating a high plasticity of the information content of the genomes. The presence of exons in many circular cellular RNAs supports the view that the HDAg and DL-Ags originated from a host organism (Kristensen et al., 2019). A host-derived protein-encoding sequence as proposed for the delta-interacting protein A (DIPA) may have been incorporated into a HDV ancestral genome (Brazas and Ganem, 1996; Long et al., 1997). For both sDLAs and rDLAs proteins, larger versions of the DL-Ags were not detected in contrast to the HDAg-S/-L versions expressed by HDV. The presence of ORFs and also regulatrory elements is not unexpected if the delta-like agents are related to circRNAs (Chen et al., 2016). Remarkably, circRNAs can be translated through different mechanisms, internal ribosome entry site (IRES) dependent or supported by base modification (Chen and Sarnow, 1995; Abe et al., 2015; Wang and Wang, 2015; Pamudurti et al., 2017; Yang et al., 2017).

(ii) HDV and Its Ribozyme

Self-cleaving RNA motifs or ribozymes play important roles for facilitating rolling circle replication of circular RNAs such as HDV RNA, viroids, or satellite RNAs. Self-cleaving ribozymes are classified based on the secondary and tertiary structures of the catalytic RNA motifs, which are unique for each family, such as hairpin, hammerhead, HDV-like and twister (Jimenez et al., 2015). HDV-like ribozymes are widespread and have been identified in retrotransposons and various genomic loci suggesting miscellaneous biological functions, possibly providing an extra level of control for expression of the genes in which they are located (Webb and Lupták, 2011; Riccitelli and Lupták, 2013). The HDV-ribozyme and related ribozymes are modeled into pseudoknotted secondary structures (Perrotta and Been, 1991; Webb and Lupták, 2011), the crystal structure of the related HDV genomic and antigenomic ribozymes revealed the presence of a nested, double pseudoknot structure (Ferré-D’Amaré et al., 1998; Wadkins et al., 1999) (Figure 3). A ribozyme with structural similarity to the HDV ribozymes was first identified in an intron of the human cytoplasmic polyadenylation element-binding protein (*CPEB3*) gene and is possibly involved in co-transcriptional processing of *CPEB3* primary RNA transcripts (Salehi-Ashtiani et al., 2006). The CPEB3 ribozyme is highly conserved among mammals, but the CPEB3 ribozyme sequences are substantially different to the HDV genomic and antigenomic ribozyme sequences. Approximately 60 nucleotides are required to form the conserved nested double-pseudoknot structure, only six nucleotides are invariant (Webb et al., 2009). Structure-based searches identified CPEB3 related sequences in non-mammalian genomes demonstrating their wide distribution. HDV-like ribozymes have been identified in all branches of life, and found in the genomes of the *Anopheles gambia*, *Drosophila* species, the insect virus Chilo iridescent virus, sea urchin *Strongylocentrotus purpuratus*, lamprey *Petromyzon marinus*, lancelet *Branchiostoma floridae*, nematodes *Caenorhabditis japonica* and *Pristionchus pacificus*, and outside of the eukaryotics in the bacterium *Faecalibacterium prausnitzii* (Webb et al., 2009; Eickbush and Eickbush, 2010; Ruminski et al., 2010). Interestingly, HDV-like ribozymes have been located at the 5′-end of retrotransposons suggesting that it represents an ancient element, possibly involved in the cotranscriptional processing of retroelements, and is spread by retrotransposition (Webb and Lupták, 2011). For instance, HDV-like ribozymes have been identified at the 5′-untranslated region of the R2 non-long terminal repeat (LTR) retrotransposon in *Drosophila* (Eickbush and Eickbush, 2010; Ruminski et al., 2010), and at the 5′-end of the L1 and NAR retrotransposons identified in *Trypanosoma cruzi* (L1Tc and NARTc, respectively) (Bringaud et al., 2002; Sánchez-Luque et al., 2012; 2011). The R2 elements are site-specific retrotransposons inserted into the 28S ribosomal RNA (rRNA) genes of most insect species, and are co-transcibed with the 28S rRNA (Moss et al., 2011). The presence of the HDV-like ribozyme in the 5′-untranslated region of the R2 allows self-cleavage of the 28S-R2 cotranscript at the junction between the 28S rRNA and the R2 element (Eickbush and Eickbush, 2010; Ruminski et al., 2010). Translation initiation of the open reading frame of the uncapped R2 transcript is possibly facilitated by the HDV-like ribozyme, which is thought to act also as an IRES (Ruminski et al., 2011). The interaction of the R2 protein with the R2 RNA to form a protein-RNA complex is required to target the 28S rDNA to faciliate insertion, which involves binding to the 3′- and 5′-sequences including the R2 pseudoknot structure at the 5′-end. The R2 elements have been active in the Drosophila lineage since the origin of the genus 50 million years ago. Comparative studies on the HDV-like ribozymes from different Drosophila species revealed considerable sequence changes. Remarkably, 21 out of 27 nucleotides of the R2 ribozyme catalytic core are the same as those in the HDV ribozyme (Eickbush and Eickbush, 2010). Similarly, the L1Tc retrotransposon of *Trypanosoma cruzi* contains a HDV-like ribozyme, which facilitates the release of the transposon from a polycistronic RNA (Sánchez-Luque et al., 2012; 2011). Interestingly, L1Tc contains a dual promoter and ribozyme system. The 77 nucleotides of the L1Tc HDV-like ribozyme also act as an internal promoter (Pr77) at the DNA level. The HDV-like ribozyme cleaves upstream of its catalytic core (Ferré-D’Amaré et al., 1998; Perrotta and Been, 1991) and hence, the regulatory sequence, which contains ribozyme and promoter is preserved in the L1Tc RNA and after transposition (Sánchez-Luque et al., 2012, 2011). Sequences downstream of the L1Tc ribozyme can induce structural changes which interfere with the ribozyme activity and may promote an conformational switch to a possible IRES structure to facilitate initiation of translation, as in the case of the R2 element (Sánchez-Luque et al., 2012). The HDV ribozyme and HDV-related ribozymes are common in diverse biological systems which are at different levels of the evolutionary ladder with suggested various biological roles supporting rolling circle replication, mRNA biogenesis and gene regulation. The HDV ribozyme plays an integral role in HDV replication to generate monomeric genomes and antigenomes. The presence of HDV-like ribozymes in specific non-LTR retrotransposons and the ability of HDV-like ribozymes to retain their intact catalytic core after cleavage may have facilitated their spread by retrotransposition. It is unclear whether the nested, double knot ribozymes result from converged evolution. The sequence is highly divergent but preserved a highly complex structure with a higher level of constraints for retaining cleavage activity compared to other classes of ribozymes (Legiewicz et al., 2006; Nehdi and Perreault, 2006). The widespread distribution of HDV-related ribozymes and their ability to provide multiple functions beyond cleavage activity strongly suggests that several factors contributed to the selection for the HDV-like ribozyme. It is most likely that the ribozyme present in the HDV RNA is derived from a host transcriptome, and then optimized for cleavage to support the efficient replication of the HDV RNAs. Although Hepadnaviruses from various mammals can provide the helper function to support HDV assembly, natural HDV infections have been so far only observed in humans. HDV possibly originated from a circular RNA molecule, probably a delta-like agent, which co-evolved with HBV in the human lineage. The discovery of delta-like agents in divergent organisms (Wille et al., 2018; Chang et al., 2019; Hetzel et al., 2019; Paraskevopoulou et al., 2020), the existence of cellular cirular RNAs, and the presence of HDV-like ribozymes in cellular transcripts points toward a cellular origin of HDV.

(iii) HDV and Viroids

Similarities between HDV and viroids have been recognized based on their single stranded circular RNAs which possess a high degree on self-complementarity. Intramolecular base-pairing allows the formation of secondary structures, rod-like structures (HDV and viroids of the family *Pospiviroidae*) or Y-shaped or branched structures (viroids of the family *Avsunviroidae*). The viroids replicate in two different cellular compartments, the nucleus for the *Pospiviroidae*, and the chloroplast for the *Asunviroidae*. HDV replication is restricted to the nucleus (Flores et al., 2016; Adkar-Purushothama and Perreault, 2019). Similar to HDV, the non-protein coding viroid RNAs attract host DNA-dependent RNA polymerases for replication, which is possibly facilitated by the rod- and branched-liked secondary structures of the RNA molecules (Table 1). Members of the *Avsunviridae* replicate by a symmetric rolling circle cycle generating (+) and complement (-) circles, similar to the HDV replication cycle producing circular genomic and antigenomic HDV RNA molecules. This is in contrast to the asymmetric replication cycle of viroids of the family *Pospiviroidae*, which generate only (+) circles followed by cleavage of oligomeric strands by a host RNase III (Flores et al., 2009). HDV RNA cleavage of the oligomeric (+) and (−) strands, or co-transcriptional self-cleavage is assisted by *cis*-acting pseudoknot-like ribozymes (Ferré-D’Amaré et al., 1998; Perrotta and Been, 1991). Structurally distinct *cis*-acting hammerhead ribozymes are present in viroids of the family *Avsunviroidae*. The secondary structures and short double strandedness of the RNA molecules may have provided an evolutionary advantage to minimize detection by double-stranded RNA-dependent protein kinases, to provide resistance against endonucleases, and to exhibit motifs for replication, and in addition for HDV, the editing site for generating the extended ORF encoding HDAg-L. HDV shares similarities with both viroid families, the rod-like secondary structure and replication in the nucleus like the members of the *Pospiviroidae*, and the symmetric replication mechanism and cis-acting ribozyme activity is shared with the *Avsunviridae*, although the ribozyme activiy is provided by differently structured ribozyme types. Sequence complementary of viroids with the cellular 7S RNA, a component of the signal recognition particle, and a corresponding sequence similarity of the HDV antigenomic RNA and its human counterpart 7SL RNA revealed additional similarities (Negro et al., 1989; Symons, 1989; Young and Hicke, 1990). Based on the similarities between viroids and HDV circles and their intramolecular base-pairing, a concept was proposed that HDV consists of two distinct domains. One domain contains the coding sequence for the delta antigen, and the second the viroid-like domain with a size of approximately 350 nucleotides with the sequences required for replication and also the self-cleaving ribozyme activity (Lai, 1995; Lafontaine et al., 1997). The presence of different ribozyme motifs specific for HDV and delta-like agents vs. *Avsunviridae* possibly indicates that HDV is not a viroid derivative generated by recombination events.

## Concluding Remarks

The findings that (i) circular RNA species are abundant in eukaryotic cells, (ii) HDV-like ribozymes are present in highly divergent organisms with important roles in many molecular pathways, and (iii) delta-like circular RNA agents are found in vertebrates and invertebrates, suggest that HDV is derived from the cellular transcriptome. Delta-like agents have not yet been identified in non-human primates strongly indicating that a transspecies transmission event of a delta-like agent into a HBV-infected human host allowed a host shift and the emergence of HDV. The HDV sequence diversity is possibly a consequence of human migration and geographic vicariance.

## Declaration

The author HJN of this publication has an equity interest in, and serves as a consultant to ClearB Therapeutics. ClearB Therapeutics had no role in the writing of the article.

## Author Contributions

All authors listed have made a substantial, direct and intellectual contribution to the work, and approved it for publication.

## Conflict of Interest

The author HJN has an equity interest in, and serves as a consultant to ClearB Therapeutics. ClearB Therapeutics had no role in the writing of the article. The remaining authors declare that the research was conducted in the absence of any commercial or financial relationships that could be construed as a potential conflict of interest.
